# Late-Onset Para-Aortic Lymph Node Recurrence 11 Years after Resection of Pancreatic Cancer: Histological Reassessment Revealed Neuroendocrine Differentiation Amenable to Surgical Resection

**DOI:** 10.70352/scrj.cr.25-0374

**Published:** 2025-09-27

**Authors:** Yohei Tabe, Daisuke Asano, Hayato Takamizawa, Satoshi Matsui, Yoshiya Ishikawa, Hiroki Ueda, Keiichi Akahoshi, Eriko Katsuta, Yuki Kato, Yuko Kinowaki, Daisuke Ban

**Affiliations:** 1 Department of Hepatobiliary and Pancreatic Surgery, Graduate School of Medicine, Institute of Science Tokyo, Tokyo, Japan; 2 Department of Professional Development, Institute of Science Tokyo Hospital, Tokyo, Japan; 3 Department of Human Pathology, Graduate School of Medicine, Institute of Science Tokyo, Tokyo, Japan; 4 Department of Comprehensive Pathology, Graduate School of Medicine, Institute of Science Tokyo, Tokyo, Japan

**Keywords:** pancreatic ductal adenocarcinoma, pancreatic neuroendocrine neoplasm, para-aortic lymph node metastasis, late recurrence, histopathological re-evaluation

## Abstract

**INTRODUCTION:**

While para-aortic lymph node recurrence may occur after curative resection for pancreatic ductal adenocarcinoma (PDAC), it is rarely seen as a late recurrence and is usually treated with systemic chemotherapy. In contrast, recurrence of pancreatic neuroendocrine neoplasms (Pan-NENs) can occur more than a decade after surgery and may be amenable to surgical resection. Although immunohistochemical techniques have significantly enhanced the diagnostic distinction between PDAC and Pan-NEN, challenges remain, particularly in older cases.

**CASE PRESENTATION:**

A 77-year-old man underwent distal pancreatectomy for pancreatic body cancer 11 years prior, initially diagnosed as PDAC based on glandular morphology, and received adjuvant S-1 chemotherapy. Surveillance ended after 5 years without recurrence. Eleven years after surgery, imaging performed during evaluation for elevated prostate-specific antigen revealed a 16-mm para-aortic lymph node enlargement. CT-guided biopsy showed neuroendocrine morphology with chromogranin A and synaptophysin positivity, consistent with neuroendocrine tumor (NET). Retrospective histological review of the original surgical specimen revealed previously unrecognized NET-G2 components (Ki-67, 5.0%). Surgical resection of the para-aortic and mesenteric lymph nodes was performed, and pathology confirmed NET-G2 metastases (Ki-67, 6.7%). The patient recovered uneventfully and remains recurrence-free at 7 months postoperatively under active surveillance.

**CONCLUSIONS:**

This case highlights the importance of tissue diagnosis when late-onset recurrence is observed in patients with a prior diagnosis of PDAC. It also underscores the potential for extremely delayed recurrence in Pan-NENs and the importance of long-term surveillance. For patients presenting with isolated distant recurrence long after initial treatment for PDAC, biopsy and pathological re-evaluation should be considered to ensure appropriate therapeutic decision-making.

## Abbreviations

EUS-TA
: endoscopic ultrasound-guided tissue acquisition
FDG
: fluorodeoxyglucose
NET
: neuroendocrine tumor
PALN
: para-aortic lymph node
Pan-NEN
: pancreatic neuroendocrine neoplasm
PDAC
: pancreatic ductal adenocarcinoma

## INTRODUCTION

Pancreatic ductal adenocarcinoma (PDAC) is one of the most aggressive malignancies; thus, we often experience a recurrence after curative resection. Typical recurrence occurs within 5 years, and the common recurrent sites are liver, peritoneal, lung, and local recurrence.^1)^ Although para-aortic lymph node (PALN) recurrence is not as common as the aforementioned metastatic sites, Hishinuma et al. reported approximately 20% of PALN metastasis in patients with PDAC recurrence based on autopsy analysis.^2)^ In such cases, systemic chemotherapy is generally the mainstay of treatment.

In contrast, pancreatic neuroendocrine neoplasms (Pan-NENs) generally carry a more favorable prognosis than PDAC. Nevertheless, recurrence after curative resection occurs in approximately 20%–30% of cases.^3)^ The most frequent recurrent site is the liver, accounting for nearly 80% of metastases. Although PALN metastasis has also been reported in Pan-NENs,^4,5)^ it remains relatively uncommon. Unlike PDAC, treatment options for distant recurrence of Pan-NEN include surgical resection, depending on factors such as tumor burden, recurrent tumor location, and biological behavior.

Although immunohistochemistry of neuroendocrine markers has greatly improved the ability to distinguish PDAC from Pan-NEN, it sometimes remains challenging. Notably, in the early 2010s, the classification of neuroendocrine tumors (NETs) was still evolving, and tumors with ambiguous histological features may have had their neuroendocrine components underrecognized or misclassified.

Here, we report a case of PALN recurrence 11 years after curative resection for a tumor initially diagnosed as PDAC. The lesion’s atypical clinical behavior prompted re-biopsy and pathological re-evaluation, which revealed previously unrecognized neuroendocrine differentiation. This case highlights the importance of histological reassessment in patients with unusual recurrence patterns, particularly in those diagnosed prior to the establishment of current diagnostic standards.

## CASE PRESENTATION

**Fig. 1:**
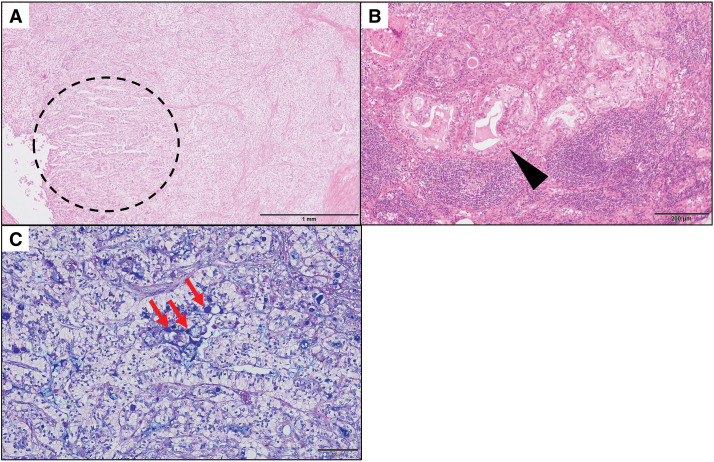
Histopathological findings at the time of distal pancreatectomy for pancreatic body cancer. (**A**) H&E staining of the primary tumor. Glandular structures are observed within the tumor area (outlined by a black dotted circle). Scale bar indicates 1 mm. (**B**) H&E staining of a metastatic lymph node. Eosinophilic secretions within the glandular lumina are evident (black arrowhead). Scale bar indicates 200 μm. (**C**) PAS–Alcian blue staining confirming the presence of mucin-containing cells (red arrows). Scale bar indicates 100 μm. H&E, hematoxylin and eosin; PAS, periodic acid Schiff

A 77-year-old man was diagnosed with a tumor in the pancreatic body and underwent distal pancreatectomy with splenectomy 11 years ago. Histopathological examination of the surgical specimen at the time of the initial surgery revealed glandular structures. Periodic acid Schiff–Alcian blue staining confirmed the presence of mucin-containing cells (**Fig. 1**), leading to a diagnosis of invasive PDAC. Based on the pathological findings, the tumor was diagnosed as pT3pN1pM0, fStage IIB according to the Union for International Cancer Control 7th edition. Postoperative adjuvant chemotherapy with S-1 was completed successfully.

**Fig. 2:**
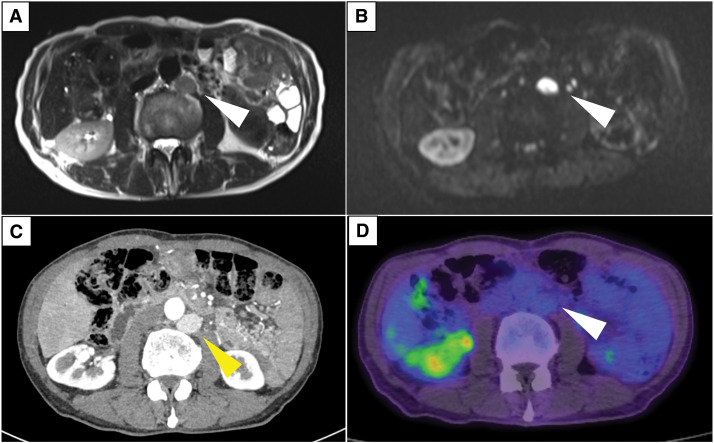
Imaging findings when PALN enlargement was detected. MRI revealed a 16-mm PALN enlargement indicated by white arrowheads. (**A**) T2-weighted image showing a hyperintense PALN. (**B**) Diffusion-weighted image demonstrating restricted diffusion in the same lesion. (**C**) Contrast-enhanced CT demonstrating the same lesion, indicated by a yellow arrowhead. (**D**) FDG PET-CT demonstrated no significant abnormal uptake in the enlarged lymph node, indicated by a white arrowhead. FDG, fluorodeoxyglucose; PALN, para-aortic lymph node

**Fig. 3:**
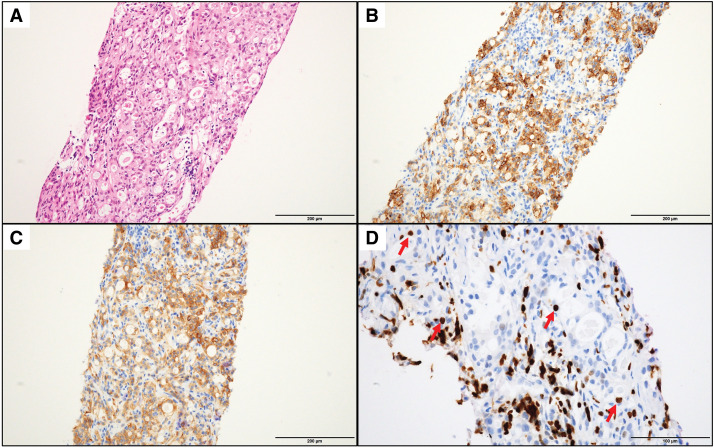
Histopathological findings of the recurrent para-aortic lymph node obtained by CT-guided biopsy. (**A**) H&E staining of the lymph node metastasis. Tumor cells are arranged in trabecular and nest-like patterns, suggestive of neuroendocrine differentiation. Scale bar indicates 200 μm. (**B**) Immunohistochemical staining for chromogranin A demonstrates strong cytoplasmic positivity in tumor cells. Scale bar indicates 200 μm. (**C**) Immunohistochemical staining for synaptophysin shows diffuse cytoplasmic positivity in tumor cells. Scale bar indicates 200 μm. (**D**) Ki-67 staining reveals a labeling index of 30.9%; however, background lymphocytes also showed nuclear positivity, and the actual index in tumor cells was estimated to be approximately 5% (indicated by red arrows). Scale bar indicates 100 μm. H&E, hematoxylin and eosin

Routine surveillance for PDAC was discontinued after 5 years with no evidence of recurrence. Eleven years after the initial surgery, during an evaluation for elevated prostate-specific antigen levels at another facility, MRI revealed a 16-mm PALN enlargement adjacent to the left renal artery (**Fig. 2**). Contrast-enhanced CT verified the enlarged PALN seen on MRI and confirmed no other evidence of tumor. Fluorodeoxyglucose PET/CT (FDG-PET/CT) demonstrated no significant abnormal uptake, including in the enlarged lymph node. Serological examinations, including tumor markers such as carcinoembryonic antigen (CEA), carbohydrate antigen 19-9, and neuron-specific enolase, revealed no remarkable abnormalities. Given the unusually late onset and isolated nature of the recurrence, which was atypical for PDAC, a CT-guided biopsy of the PALN was performed. Initial histological examination revealed tumor cells positive for chromogranin A and synaptophysin, with a Ki-67 labeling index of 30.9%, initially suggestive of NET-G3 according to the World Health Organization (WHO) 2019 classification. However, closer inspection revealed that background lymphocytes also exhibited nuclear Ki-67 positivity, and the true proliferative index within the tumor cells was estimated to be approximately 5% (**Fig. 3**). Following these findings, somatostatin receptor scintigraphy was undertaken but demonstrated no significant uptake.

**Fig. 4:**
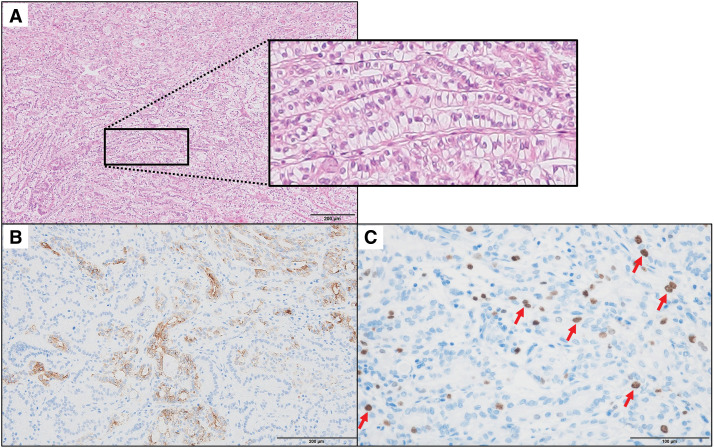
Re-evaluation of the initial surgical specimen. (**A**) H&E staining showing cord-like structures suggestive of neuroendocrine differentiation. A magnified image of the cord-like structures is shown on the right. Scale bar indicates 200 μm. (**B**) Immunohistochemical staining for chromogranin A demonstrating focal (< 30%) cytoplasmic positivity in tumor cells. Scale bar indicates 200 μm. (**C**) Ki-67 staining showing a labeling index of 5.0%, consistent with a diagnosis of NET-G2. Red arrows indicate tumor cells. Scale bar indicates 100 μm. H&E, hematoxylin and eosin; NET, neuroendocrine tumor

Given these unexpected findings, a retrospective histopathological review of the archival surgical specimen from the initial surgery was performed. Detailed examination revealed scattered cord-like structures suggestive of neuroendocrine differentiation in a small part of the PDAC tumor. Because morphological evaluation alone was insufficient for a definitive diagnosis, additional immunohistochemical staining was performed, revealing focal positivity (< 30%) for chromogranin A and synaptophysin, with a Ki-67 labeling index of 5.0%, consistent with NET-G2 (**Fig. 4**). As the neuroendocrine component accounted for less than 30% of the total tumor volume, the diagnosis of mixed neuroendocrine–non-neuroendocrine neoplasm was excluded in accordance with the WHO 2019 classification, which requires both components to exceed 30%. Based on this re-evaluation, the initial tumor was retrospectively diagnosed as PDAC with a neuroendocrine component.

**Fig. 5:**
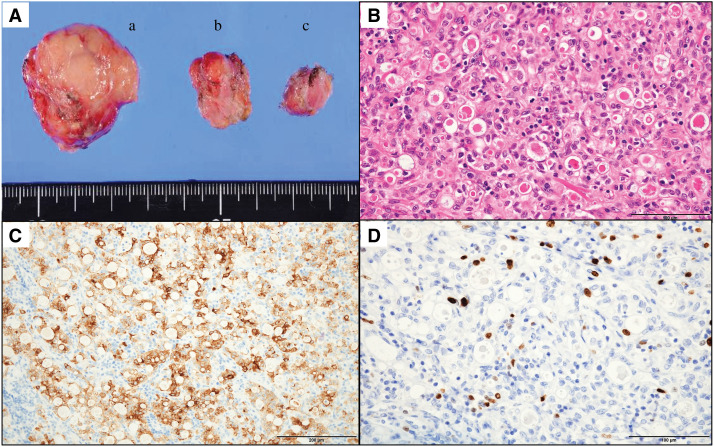
Histopathological findings of the resected lymph nodes. (**A**) Macroscopic photograph of the resected lymph nodes. (a) Para-aortic lymph node; (b, c) colonic mesenteric lymph nodes. (**B**) H&E staining of the para-aortic lymph node, showing glandular and nest-like structures suggestive of neuroendocrine differentiation. Scale bar indicates 100 μm. (**C**) Immunohistochemical staining for chromogranin A demonstrating diffuse cytoplasmic positivity in tumor cells. Scale bar indicates 200 μm. (**D**) Ki-67 staining showing a labeling index of 6.7%, consistent with a diagnosis of NET-G2. Scale bar indicates 100 μm. H&E, hematoxylin and eosin; NET, neuroendocrine tumor

Based on these findings, we concluded that the PALN lesion was a metastasis originating from the NET component of the pancreatic tumor. Given the lesion’s indolent progression and solitary presentation, open surgical resection of the PALN was undertaken. Two additional enlarged mesenteric lymph nodes were also identified intraoperatively and subsequently resected. Histopathological examination of the 3 lymph nodes confirmed metastases of NET-G2 with a Ki-67 index of 6.7% (**Fig. 5**).

The postoperative course was uneventful, and monthly lanreotide treatment was administered as adjuvant therapy for 6 months based on the clinical judgment of the attending physician. At the 7-month follow-up, the patient remained disease-free under active surveillance.

## DISCUSSION

This case represents an unusual instance of PALN recurrence occurring 11 years after resection of a tumor initially diagnosed as PDAC. The lesion exhibited an atypical clinical course for PDAC recurrence, including the extremely late onset, lack of FDG uptake, and absence of other metastatic disease. These atypical features prompted a tissue biopsy to achieve a definitive diagnosis before initiating systemic therapy. CT-guided biopsy was chosen due to the lesion’s location beyond the reach of endoscopic ultrasound-guided tissue acquisition (EUS-TA), although, when feasible, EUS-TA offers comparably high sensitivity and accuracy.^6)^

PDAC is known for early recurrence, typically within 2 years following resection.^7)^ On the other hand, Pan-NENs exhibit variable recurrence patterns. While most recurrences occur within 5 years after surgery, multiple studies have documented recurrence even after a decade. For example, Yao et al. reported that delayed recurrence beyond 10 years is possible in Pan-NENs, highlighting their potential for prolonged dormancy.^8)^ In our institutional cohort of 145 patients who underwent curative resection for localized Pan-NETs between January 2014 and September 2023, postoperative recurrence occurred in 9 cases (6.2%), with 2 of these cases presenting recurrence more than 5 years after surgery (**Table 1**).

**Table 1:** Cases with postoperative recurrence

	Age	Sex	RFS (months)	OS (months)	Recurrence site	Ki-67 index (%)	WHO grade	Status
1	49	Female	14.4	114.7	Liver	1.4	G1	Alive
2	45	Male	17.7	81.1	Lymph node	12.7	G2	Alive
3	46	Female	21.3	73.5	Liver	1.5	G1	Alive
4	59	Male	26.2	76.9	Liver	27.5	G3	Alive
5	81	Female	28.4	58.3	Liver	9.4	G2	Alive
6	62	Male	33.6	91.7	Liver	2.6	G1	Alive
7	64	Male	42.6	93.7	Liver	3.6	G2	Alive
8	65	Male	86.4	116.1	Liver	1.0	G1	Alive
9	71	Male	91.1	92.5	Liver	7.5	G2	Alive

Age indicates the patient’s age at the time of surgery. WHO grade was assigned based on the WHO 2019 classification of neuroendocrine tumors.

OS, overall survival; RFS, recurrence-free survival; WHO, World Health Organization

Management of distant recurrence in Pan-NENs, including liver and lymph node metastases, is often debated. However, several reports have shown that in cases with isolated metastases, surgical resection can offer durable disease control. Mayo et al. demonstrated improved survival in patients undergoing resection of hepatic metastases.^9)^ In the context of lymph node recurrence, Hane et al.^4)^ and Kishimoto et al.^5)^ reported long-term survival exceeding 7–10 years after resection of PALN metastases. Although the observation period is limited, our case also demonstrates a disease-free course following surgery, with no evidence of recurrence at 7 months postoperatively. According to the National Comprehensive Cancer Network guidelines, follow-up includes assessment, markers, and imaging at 12 weeks to 12 months after resection, then every 6–12 months for 10 years. Given the high risk of recurrence associated with distant lymph node metastasis, we consider a follow-up schedule of imaging every 3 months in the 1st year, every 6 months from the 2nd through the 5th year, and annually thereafter up to 10 years, with extension as clinically indicated.

This case also underscores the diagnostic complexity of differentiating PDAC from Pan-NENs with atypical morphology. The pancreatic tumor was diagnosed as adenocarcinoma based on glandular architecture and mucin production, and the lesional lymph node metastasis was predominantly composed of adenocarcinoma elements at the initial pathological diagnosis. In addition, imaging showed a hypovascular mass with ill-defined margins and an associated pancreatic duct stricture, which contrasts with the typical NET appearance of a well-circumscribed, hypervascular lesion. Therefore, NET was not considered in the differential diagnosis at the time, and immunohistochemical staining for neuroendocrine markers was not performed. However, on retrospective review, additional immunohistochemical staining revealed focal positivity for chromogranin A and synaptophysin, with a Ki-67 labeling index of 5.0%, consistent with NET-G2. This likely reflects both the limited use of immunohistochemical staining and the relatively low clinical recognition of NETs in the early 2010s,^10)^ which made accurate histopathological differentiation more difficult. We now explicitly note that older cases—diagnosed years earlier—may warrant immunohistochemical re-evaluation using modern techniques, as this can reveal previously unrecognized tumor components and impact management.

Upon retrospective review, a portion of the tumor exhibited features consistent with NET-G2. Taking the above observations into consideration, it is likely that the adenocarcinoma component was completely eradicated by surgical resection followed by adjuvant S-1 chemotherapy, whereas the residual NET component persisted, causing delayed recurrence. Another possibility is that the lesion represented a *de novo* NET in the lymph node; however, this is considered extremely unlikely, as comprehensive imaging studies revealed no evidence of another primary site. Recognition of the NET component at the outset would not have altered the adjuvant treatment, as S-1 remains standard for PDAC and no proven adjuvant therapy exists for NETs.

Another important consideration in this case is the discrepancy between Ki-67 indices obtained from the biopsy specimen (30.9%) and the resected surgical specimens (6.7%). One possible explanation is that reactive lymphoid proliferation in the biopsy may have led to an overestimation of the Ki-67 index. In daily practice, such discrepancies are not uncommon, particularly in small biopsy samples, because it can be difficult to determine whether Ki-67-positive nuclei belong to tumor cells or inflammatory cells.^11)^ In the present case, surgical resection was undertaken due to the lesion’s indolent progression. However, if the lesion had been definitively classified as NET-G3, a more conservative approach to surgical intervention may have been considered.^12)^ This highlights the importance of meticulous tumor grading to optimize treatment planning.

## CONCLUSIONS

We report a rare case of PALN recurrence of Pan-NEN occurring 11 years after initial resection, which had originally been diagnosed as PDAC. This case highlights an important clinical consideration: in patients with a prior diagnosis of PDAC who present with atypical patterns of recurrence, tissue biopsy and histopathological reassessment should be strongly considered before initiating systemic therapy. Such diagnostic vigilance may reveal previously unrecognized neuroendocrine components, enabling potentially curative surgical intervention. Ultimately, this case underscores the importance of individualized management, long-term surveillance, and critical re-evaluation in the setting of unexpected recurrence.

## DECLARATIONS

### Availability of data and materials

The data that support the findings of this study are available from the corresponding author upon reasonable request.
